# The Comparison of Therapeutic With Prophylactic Anticoagulation on Mortality, Risk of Bleeding, and Thromboembolism in Hospitalized Patients With COVID-19: A Systematic Review

**DOI:** 10.7759/cureus.29932

**Published:** 2022-10-05

**Authors:** Nang I Kham, Rabia Shahid, Shaili S Naik, Shivana Ramphall, Swarnima Rijal, Vishakh Prakash, Heba Ekladios, Jiya Mulayamkuzhiyil Saju, Naishal Mandal, Prachi Balani

**Affiliations:** 1 Internal Medicine, California Institute of Behavioral Neurosciences and Psychology, Fairfield, USA; 2 Hospital Medicine, University of Medicine 1, Yangon, MMR; 3 Internal Medicine, Surat Municipal Institute of Medical Education and Research (SMIMER) Hospital and Medical College, Surat, IND; 4 Research, American University of Antigua, Osbourn, ATG; 5 Internal Medicine, Government Medical College, Kozhikode, IND; 6 Psychiatry, California Institute of Behavioral Neurosciences and Psychology, Fairfield, USA; 7 General Surgery, Government Medical College, Trivandrum, IND; 8 Internal Medicine, Sree Narayana Institute of Medical Sciences, Ernakulam, IND; 9 Internal Medicine, Saint Vincent Hospital, Worcester, USA

**Keywords:** risk of covid-19 mortality, arteriovenous thromboembolism in sars-cov-2-infected patients, critical hemorrhagic complications, prophylactic anticoagulation, therapeutic anticoagulation, coronavirus disease 2019 (covid-19)

## Abstract

Thromboembolism is one of the most severe manifestations of coronavirus disease 2019 (COVID-19). Thrombotic complications have been reported even with the administration of thromboprophylaxis. This has led many experts to have variable opinions on the most effective prophylactic strategy and to anticipate the discovery of the ideal dosing of anticoagulation to reduce thromboembolic events and related mortality. We performed a systematic review to evaluate whether therapeutic-dose anticoagulation is superior to prophylactic-dose anticoagulation by comparing mortality rates, bleeding risks, and rates of thromboembolism. We adhered to the Preferred Reporting Items for Systematic Reviews and Meta-Analyses (PRISMA) guidelines to create our systematic review. Twenty-two records were collected from PubMed, PubMed Central (PMC), and Medical Literature Analysis and Retrieval System Online (MEDLINE), after which they undertook quality appraisals. A total of 124 studies were analyzed in six systematic reviews and meta-analyses, one pooled analysis, two multicenter retrospective cohort studies, one observational study, one retrospective chart review, one evidence-based protocol, and four narrative reviews.

## Introduction and background

The coronavirus disease 2019 (COVID-19) has impacted over 560 million individuals worldwide, resulting in over six million fatalities from its first incidence in December 2019 in China to July 2022 globally [1]. Although severe acute respiratory syndrome coronavirus 2 (SARS-CoV-2) predominantly affects the respiratory system, it could also impair other organ systems [2]. The pathophysiology and severity of COVID-19 mainly depend on the degree to which the immune and inflammatory systems are compromised, in addition to direct viral effects. In severe cases, over-accumulating pro-inflammatory cytokines promote hypercoagulation, vascular hyperpermeability, multiorgan failure, and even death. These findings support the increased reports of venous thromboembolism (VTE) and pulmonary embolism (PE) in severe cases of COVID-19, which are considered poor prognostic indicators [2-5]. Consequently, elevated D-dimer, fibrinogen, and indicators of endothelial dysfunction (von Willebrand factor antigen and soluble thrombomodulin) have been associated with unfavorable prognoses, including death [5,6]. As a result, several clinical trials have emerged and initiated studies designed to improve COVID-19 prognosis with antithrombotic treatment [7]. The incorporation of thromboprophylaxis in hospitalized COVID-19 patients has been found to increase survival [8].

On top of its antithrombotic action, heparin exerts an anti-inflammatory effect, a protective action on endothelial function [9], and a specific antiviral action in the extracellular matrix of tissues [10]. A short retrospective cohort analysis showed that the administration of low-molecular-weight heparin (LMWH) resulted in an increased number of lymphocytes and lower interleukin-6 (IL-6) levels compared to control patients, demonstrating an improvement in coagulation profiles and the restoration of immune functions. In another study, heparin treatment was related to increased oxygenation in 27 individuals with COVID-19 [2]. Despite the administration of thromboprophylaxis, COVID-19 patients are more likely to develop VTE than severely ill COVID-19-negative patients. This occurrence has prompted many experts to propose higher anticoagulant dosages, particularly in patients who are at increased risk of thromboembolism due to significantly elevated D-dimer results and/or other comorbidities [5]. Meanwhile, the most efficient thromboprophylactic strategies for a wide range of COVID-19 disease severity are still undetermined [11]. According to current national and international recommendations, hospitalized patients are indicated for universal pharmacologic thromboprophylaxis with subcutaneous LMWH or unfractionated heparin (UFH) [12]. The National Institutes of Health (NIH) guidelines recommend the use of a therapeutic dose of heparin for nonpregnant patients with D-dimer levels above the upper limit of normal who require conventional oxygen and do not have an increased bleeding risk unless heparin is contraindicated. For other patients, including pregnant females, the panel recommends the prophylactic dose of heparin if there is no contraindication [13].

The lack of consensus about the ideal prophylactic anticoagulant regimen has resulted in diverse expert advice, hospital policies, and clinical judgments regarding various antithrombotic treatment modalities [3]. In continuing randomized controlled trials (RCTs) emphasizing outpatients, hospitalized patients in medical wards, and critically compromised patients with COVID-19, a number of different antithrombotic substances, dosages, and the duration of therapy are being evaluated to establish the optimal thromboprophylactic regimens [11]. We conducted a systematic review to explore whether therapeutic-dose antithrombotic therapy has a beneficial effect over prophylactic-dose antithrombosis in terms of inpatient mortality, risk of bleeding, and venous thromboembolism.

## Review

Methods

For our systematic review, we followed the Preferred Reporting Items for Systematic Reviews and Meta-Analyses (PRISMA) criteria [14]. We collected data by searching electronic databases such as PubMed, PubMed Central (PMC), and Medical Literature Analysis and Retrieval System Online (MEDLINE). We analyzed the database by using terms of Medical Subject Heading (MeSH) and keywords “anticoagulation,” “anticoagulant,” “antithrombosis,” “antithrombotic,” “thromboprophylaxis,” “prophylactic anticoagulation,” “therapeutic anticoagulation,” “heparin,” “unfractionated heparin,” “UFH,” “LMWH,” “low molecular weight heparin,” “fondaparinux,” “enoxaparin,” “COVID-19,” “Coronavirus disease 2019,” “2019 novel coronavirus,” “2019-nCO V disease,” “SARS-Co-V-2 OR SARS2,” “Severe acute respiratory syndrome coronavirus 2,” “Wuhan coronavirus,” “Alpha (B.1.1.7),” “B.1.351,” “Delta AY.4.2,” “Delta (B.1.617.2),” “Omicron (BA.1),” and “(BA.2)” separately and in combination to find relevant studies. We found 4,485 articles. We conducted a nonautomated search of the reference list of studies to identify relevant articles. Table 1 demonstrates the detailed search strategy.

**Table 1:** Designation of the search strategy UFH: unfractionated heparin; LMWH: low-molecular-weight heparin; PMC: PubMed Central; COVID-19: coronavirus disease 2019, 2019 nCO V disease: 2019 novel coronavirus disease; SARS-Co-V-2: severe acute respiratory syndrome coronavirus 2; SARS2: severe acute respiratory syndrome 2

Search strategy	Database	Number of articles before inclusion/exclusion criteria	Number of articles after inclusion/exclusion criteria
Anticoagulation OR Anticoagulant OR Antithrombosis OR Antithrombotic OR Thromboprophylaxis OR Prophylactic anticoagulation OR Therapeutic anticoagulation OR Heparin OR Unfractionated heparin OR UFH OR LMWH OR low molecular weight heparin OR fondaparinux OR Enoxaparin OR (“Anticoagulants/administration and dosage” {Majr} OR “Anticoagulants/therapeutic use” {Majr}) OR (“Heparin, Low-Molecular-Weight/administration and dosage” {Majr} OR “Heparin, Low-Molecular-Weight/therapeutic use” {Majr}) OR (“Heparin/administration and dosage” {Majr} OR “Heparin/therapeutic use” {Majr}) OR (“Enoxaparin/administration and dosage” {Majr} OR “Enoxaparin/therapeutic use” {Majr}) OR (“Fondaparinux/administration and dosage” {Majr} OR “Fondaparinux/therapeutic use” {Majr})	PubMed, PMC, and MEDLINE	33,191	3,743
COVID-19 OR Coronavirus disease 2019 OR 2019 novel coronavirus OR 2019-nCO V disease OR SARS-Co-V-2 OR SARS2 OR Severe acute respiratory syndrome coronavirus 2 OR Wuhan coronavirus OR Alpha (B.1.1.7) OR B.1.351 OR Delta AY.4.2 OR Delta (B.1.617.2) OR Omicron (BA.1) and (BA.2) OR “SARS-CoV-2/pathogenicity” (Majr)	PubMed, PMC, and MEDLINE	1,380	371
Anticoagulation OR Anticoagulant OR Antithrombosis OR Antithrombotic OR Thromboprophylaxis OR Prophylactic anticoagulation OR Therapeutic anticoagulation OR Heparin OR Unfractionated heparin OR UFH OR LMWH OR low molecular weight heparin OR fondaparinux OR Enoxaparin OR (“Anticoagulants/administration and dosage” {Majr} OR “Anticoagulants/therapeutic use” {Majr}) OR (“Heparin, Low-Molecular-Weight/administration and dosage” {Majr} OR “Heparin, Low-Molecular-Weight/therapeutic use” {Majr}) OR (“Heparin/administration and dosage” {Majr} OR “Heparin/therapeutic use” {Majr}) OR (“Enoxaparin/administration and dosage” {Majr} OR “Enoxaparin/therapeutic use” {Majr}) OR (“Fondaparinux/administration and dosage” {Majr} OR “Fondaparinux/therapeutic use” {Majr}) AND COVID-19 OR Coronavirus disease 2019 OR 2019 novel coronavirus OR 2019-nCO V disease OR SARS-Co-V-2 OR SARS2 OR Severe acute respiratory syndrome coronavirus 2 OR Wuhan coronavirus OR Alpha (B.1.1.7) OR B.1.351 OR Delta AY.4.2 OR Delta (B.1.617.2) OR Omicron (BA.1) and (BA.2) OR “SARS-CoV-2/pathogenicity” (Majr)	PubMed, PMC, and MEDLINE	1,380	371

Inclusion Criteria

We identified studies in English, randomized controlled trials, clinical trials, multicenter studies, meta-analyses, systematic reviews, traditional reviews, and evidence-based protocols on patients hospitalized with COVID-19. We incorporated the studies published after November 2019.

Exclusion Criteria

We excluded gray literature, books, documents, case series, case reports, cross-sectional studies, duplicate studies, overlapping studies, and studies before December 2019 and COVID-19 patients not requiring hospitalization.

Results

We identified a total of 4,485 articles after searching the databases (PubMed, PMC, and MEDLINE) by using the keywords and applying inclusion and exclusion criteria. After removing 708 duplicates, we screened the remaining 3,777 records by title and abstract. We assessed the derived 22 articles for quality appraisals and retained 22 reports.

**Figure 1:**
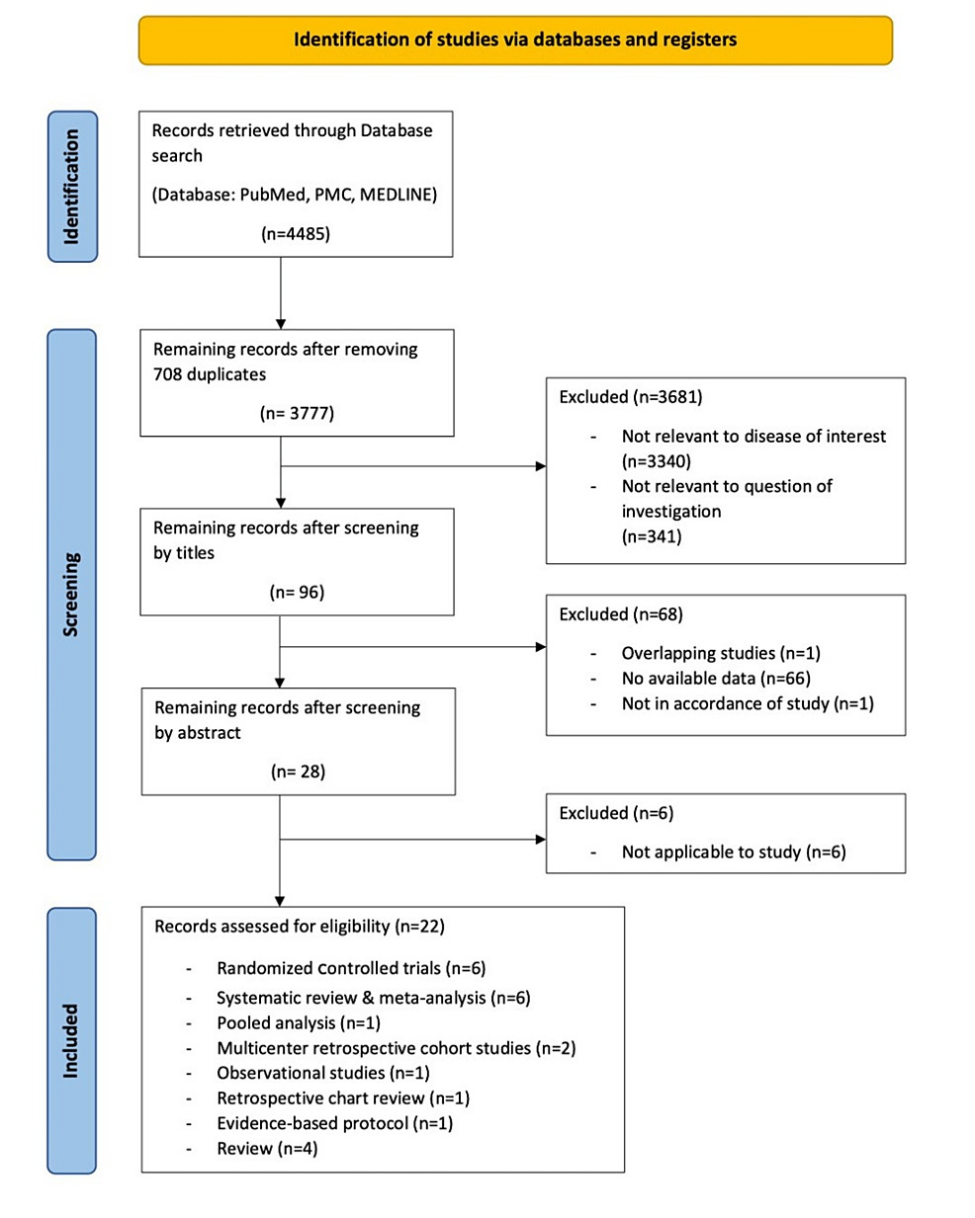
PRISMA flow diagram demonstrating the methodology PRISMA: Preferred Reporting Items for Systematic Reviews and Meta-Analyses; PMC: PubMed Central; MEDLINE: Medical Literature Analysis and Retrieval System Online

Our review included 124 studies from six randomized controlled trials (RCTs), six systematic reviews and meta-analyses, one pooled analysis, two multicenter retrospective cohort studies, one observational study, one retrospective chart review, one evidence-based protocol, and four narrative reviews with a combination of 57,100 patients. We compared the outcomes of therapeutic antithrombosis with that of regular thromboprophylaxis concerning mortality, risk of major bleeding, and thromboembolism in patients with COVID-19. The sample included in our study was a mixed population of moderate to severely ill hospitalized COVID-19 patients. The term “high-intensity anticoagulation” would be referred to as “high-dose, therapeutic-dose anticoagulation.” The term “low-intensity anticoagulation” would be defined as “prophylactic-dose, standard-dose, low-dose, intermediate-dose, usual-care, or routine anticoagulation.” A detail of our search strategy can be seen in the PRISMA flow chart (Figure 1) with reasons for excluding the primarily identified records. We compared nine studies (five RCTs and four observational studies), which included a total of 9,517 patients. Of the patients, 5,125 received low-intensity anticoagulation; 4,753 patients received high-intensity anticoagulation; and 361 patients did not receive anticoagulation. We further reviewed 13 articles that additionally covered four RCTs, 85 observational studies, and one case-control study. We used revised Cochrane risk-of-bias tool for randomized trials (RoB 2) [15] for quality appraisal of the included RCTs. We pooled the summary of nine studies in Table 2.

**Table 2:** Summary of compared studies LMWH: low-molecular-weight heparin; IU: international unit; N/A: not applicable; RCT: randomized controlled trial; UFH: unfractionated heparin

Author and year of publication	Study	Patients	Comparator	Outcomes		
				Mortality, n (%)	Major bleeding, n (%)	Thromboembolism, n (%)
Al-Banaa et al., 2022 [5]	Observational	578	Low intensity (n=131): subcutaneous LMWH 40 mg once daily or unfractionated heparin 5000 IU twice or three times daily	50 (38.2%)	1 (0.8%)	3 (2.3%)
			High intensity (n=447): subcutaneous enoxaparin 1 mg/kg twice daily or 1.5 mg/kg daily or a continuous intravenous infusion of unfractionated heparin	192 (43%)	7 (1.6%)	0
Hoogenboom et al., 2022 [16]	Observational	311	Low intensity (n=158): subcutaneous heparin or enoxaparin 40 mg twice daily	44 (28%)	N/A	4 (2.5%)
			High intensity (n=153): any heparin drip or apixaban, rivaroxaban, dabigatran, or warfarin at a typical therapeutic-dose strength or enoxaparin 1 mg/kg twice daily or 1.5 mg/kg daily	73 (49%)	N/A	23 (15%)
Sholzberg et al., 2021 [17]	RCT	465	Low intensity (n=237): prophylactic-dose LMWH or UFH	18 (7.6%)	4 (1.7%)	6 (2.5%)
			High intensity (n=228): therapeutic-dose LMWH or UFH	4 (1.8%)	2 (0.9%)	2 (0.9%)
REMAP-CAP, ACTIV-4a, and ATTACC Investigators et al., 2021 [18]	RCT	1,207 critically ill patients	Low intensity (n=564): standard low-dose or enhanced intermediate-dose anticoagulation	200/564 (35.5%)	13 (2.3%)	62 (11.1%)
			High intensity (n=534): therapeutic-dose anticoagulation	199/534 (37.3%)	20 (3.8%)	38 (7.2%)
ATTACC, ACTIV-4a, and REMAP-CAP Investigators et al., 2021 [19]	RCT	2,219 noncritically ill patients	Low intensity (n=1048): low- or intermediate-dose thromboprophylactic drugs	86 (8.2%)	9 (0.9%)	22 (2.1%)
			High intensity (n=1171): therapeutic or subtherapeutic heparin	86 (7.3%)	22 (1.9%)	13 (1.1%)
Lopes et al., 2021 [20]	RCT	615	Low intensity (n=304): prophylactic standard in-hospital enoxaparin or unfractionated heparin	23 (8%)	7 (2%)	30 (10%)
			High intensity (n=311): in-hospital oral rivaroxaban (20 mg or 15 mg daily) for stable patients or initial subcutaneous enoxaparin (1 mg/kg twice per day) or intravenous unfractionated heparin (to achieve a 0.3-0.7 IU/mL anti-Xa concentration) for clinically unstable patients, followed by rivaroxaban to day 30	35 (11%)	26 (8%)	23 (7%)
INSPIRATION Investigators et al., 2021 [3]	RCT	562	Low intensity (n=286): enoxaparin 40 mg daily	117 (40.9%)	4 (1.4%)	11 (3.8%)
			Intermediate intensity (n=276): intermediate dose (enoxaparin 1 mg/kg daily)	119 (43.9%)	7 (2.5%)	8 (2.9%)
Ionescu et al., 2021 [6]	Retrospective cohort	3,480	No anticoagulation (n=361)	11.4%	20 (5.5%)	N/A
			Low intensity (n=2121): prophylactic-dose enoxaparin, UFH, and fondaparinux	10.8%	46 (2.2%)	N/A
			High intensity (n=998): therapeutic-dose enoxaparin, UFH, fondaparinux, apixaban, warfarin, rivaroxaban, and dabigatran	23.6%	81 (8.1%)	N/A
Lemos et al., 2020 [21]	RCT	20	Low intensity (n=10): prophylactic-dose UFH or LMWH	5 (50%)	2 (20%)	N/A
			High intensity (n=10): therapeutic dose (enoxaparin 1 mg/kg twice daily)	2 (20%)	4 (40%)	N/A

Discussions

To achieve the most favorable outcomes with pharmacologic thromboprophylaxis, the benefits of intensive thromboprophylaxis must be weighed against the possible adverse outcomes [4].

Effects of Therapeutic Anticoagulation on Mortality

Many studies revealed that patients with COVID-19 receiving anticoagulation (either prophylactic or low dose; oral, subcutaneous, or intravenous) were associated with a significant reduction in mortality rates [2,22,23]. A multicenter retrospective cohort study reported that unadjusted inpatient mortality rates were comparable between high and low doses of anticoagulant groups. After adjusting for other characteristics associated with mortality, administering a high dose of anticoagulation was independently associated with decreased in-hospital mortality. The association remains consistent in sensitivity analyses, Cox regression analysis (Cox proportional hazards regression analysis), and 21-day mortality [5]. This result was in line with Ionescu et al., who said that anticoagulation therapy was correlated to lower mortality risk in the propensity score-weighted multivariate proportional hazard model. The outcome was dose-related; compared to no thromboprophylaxis, prophylactic dosing was significantly associated with a 65% decline in mortality risk and therapeutic anticoagulation with an 86% reduction [6]. According to a study evaluated by Kamel et al., giving therapeutic dosages of heparin for seven or more days was related to a better prognosis in patients with severe infection who meet sepsis-induced coagulopathy score criteria or with significantly high D-dimer [2].

The three large, open-label, adaptive, multiplatform, randomized clinical trials (REMAP-CAP, ACTIV-4a, and ATTACC) investigated the outcomes of therapeutic anticoagulation in patients hospitalized due to COVID-19. The investigators published a report for 1,098 critically ill patients receiving intensive care unit (ICU)-level support [18] and another report for 2,219 patients not initially receiving ICU-level support [19]. The latter study revealed that compared to prophylactic anticoagulation, therapeutic anticoagulation prolonged survival until hospital discharge, free from organ support in noncritically ill patients [5,19,22]. The trial stopped after the superiority of therapeutic anticoagulation was observed [19]. The former study concluded that therapeutic anticoagulation was not associated with increased survival until discharge or reduced duration of organ support compared to standard-dose anticoagulation in critically ill patients. The data safety and monitoring boards halted the trials owing to the futility of the endpoint of the freedom of organ support at 21 days and the future harmful effects of bleeding [11,18]. The inconsistent outcomes between the two studies could be explained by possible advanced pathologic conditions existing in severely ill patients to profit from therapeutic heparin [22]. Al-Banaa et al. elaborated that the difference in treatment response identified between the two studies implies that the time of initiating therapeutic anticoagulation therapy possesses a significant role in determining treatment outcomes [5]. Although the information on the timing of thromboprophylaxis administration regarding the onset of symptoms was lacking in most included studies, overall results favor the early beginning of higher dosing of thromboprophylaxis before the emergence of critical illness in patients with unfavorable prognostic features [5,8].

A meta-analysis of eight RCTs by Kow et al. concluded that there was no statistically significant difference in the death rates between high-intensity anticoagulant and low-dose anticoagulant groups in COVID-19 patients. At the same time, the author mentioned that high anticoagulant doses would be beneficial, especially in severe cases [24]. According to a meta-analysis by Kollias et al., including 17 studies (two randomized and 15 observational studies) focused on therapeutic-dose and prophylactic-dose anticoagulation, the administration of therapeutic dose did not significantly reduce the death rate compared to prophylactic dose [8]. When Kollias et al. performed a meta-analysis of three studies investigating patients solely in the ICU, the pooled adjusted relative risk (RR) for mortality in patients with COVID-19 who received a therapeutic dosage of thromboprophylaxis versus a usual-care dose was 0.58 (95% confidence interval (CI): 0.35-0.94). The author further uncovered the indication bias in the included observational studies since participants with greater risk for severe disease were more likely to receive higher doses [8]. As a trend toward clinical benefits of therapeutic anticoagulation was recognized, higher doses can be selectively recommended on an individualized basis for patients at high or very high thrombotic risk, provided they also have a low risk of bleeding [6,8,24].

Lopes et al. contradicted this statement in the open-label, multicenter anticoagulation coronavirus (ACTION) trial by disclosing that severely ill patients admitted to the ICU did not benefit from intensive or intermediate doses of antithrombotic therapy compared to prophylactic doses [20]. Sholzberg et al. speculated about the lack of anti-inflammatory and antiviral properties of heparin in rivaroxaban experimented in the ACTION trial. Moreover, using rivaroxaban and enoxaparin and including the intermediate dose in the control group could have resulted in varying results [17]. In contrast, Giossi et al. suggested that conventional thromboprophylaxis should be the initial therapy in moderate and severe COVID-19 patients, specifically with a high risk of bleeding [23]. A meta-analysis by Giossi et al. found a comparable reduction in mortality between therapeutic anticoagulation and prophylactic anticoagulation, while the prophylactic-dose cohort showed a significantly decreased risk of overall bleeding [23]. Another retrospective observational study of 311 critically ill COVID-19 patients at Stony Brook University Hospital demonstrated no significant difference in survival curves for prophylactic and therapeutic dosing domains within the first three weeks [16]. In contrast to Al-Banaa et al., this study also reported that more than three weeks of treatment with therapeutic anticoagulation increased the risk of death up to five times compared to routine anticoagulation. Hoogenboom et al. clarified that patients from the therapeutic dosing arm had a higher rate of chronic obstructive pulmonary disease (COPD); high D-dimer, lactate dehydrogenase (LDH), and brain natriuretic peptide (BNP) levels; low lymphocyte count, and partial pressure of oxygen (PaO_2_). In addition, the characteristics of non-survivors were elderly males with depleted lymphocyte counts and cardiovascular diseases. The author interpreted that variations in sample characteristics and COVID-19 disease severity may account for discrepancies in research results [16].

Intermediate vs. Standard-Dose Prophylactic Anticoagulation in Critically Ill Patients With COVID-19: An Open-Label Randomized Controlled (INSPIRATION) trial (N=562 ICU patients) demonstrated no significant differences in the primary outcome (a composite of venous or arterial thrombosis, treatment with extracorporeal membrane oxygenation, or mortality within 30 days) between both arms [3,22]. Kollias et al. mentioned that microvascular thrombi might already exist in critically ill patients, leaving increased dosing of anticoagulation ineffective [8]. The Therapeutic Anticoagulation versus Standard Care as a Rapid Response to the COVID-19 Pandemic (RAPID) trial, including 465 randomized moderately ill COVID-19 patients, did not detect a significant lowering in the 28-day composite of death, invasive mechanical ventilation, noninvasive mechanical ventilation, or ICU admission in the therapeutically anticoagulated patients; however, the occurrence of all-cause mortality was lowered by 78% in the therapeutic counterpart [17,21,22]. One study reviewed by Reis et al. concluded that therapeutic-dose anticoagulation might have little or no impact on deteriorating clinical outcomes within 28 days, as measured by advancing to intubation or death compared to conventional thromboprophylaxis. Yet, another study reviewed by the same author showed a reduction in progression to mechanical ventilation or death with therapeutic anticoagulation [7]. The Therapeutic versus prophylactic anticoagulation for severe COVID-19: A randomized phase II clinical trial (HESACOVID), a single-center study of 20 patients, published that therapeutic-dose anticoagulation significantly raised partial pressure of oxygen/fraction of inspired oxygen (PaO_2_/FiO_2_) and long ventilator-free days compared to standard-dose anticoagulation [11,25]. The association between anticoagulant dosage and mortality remains ambiguous, given the unadjusted estimates generated by varying outcomes, and should be evaluated with caution [2].

A large, prospective, multicenter, open-label, randomized controlled comparative safety and efficacy research is funded by the Icahn School of Medicine at Mount Sinai (the FREEDOM COVID-19 Anticoagulation trial) [22]. The trial is expected to enroll up to 3,600 patients, randomly assigned to one of three anticoagulation therapies (prophylactic enoxaparin, therapeutic-dose enoxaparin, or therapeutic-dose apixaban) in a 1:1:1 fashion. It is anticipated that the FREEDOM COVID-19 Anticoagulation trial is powerful enough to detect significant differences in outcomes [22]. The results from the FREEDOM COVID-19 Anticoagulation trial and other ongoing studies are crucial in inferring whether therapeutic-dose anticoagulation is superior in minimizing thrombotic events, preventing intubation, or prolonging survival compared to prophylactic-dose anticoagulation in hospitalized patients with COVID-19. Table 3 summarizes the included studies on the mortality rates of patients receiving different doses of anticoagulation.

**Table 3:** Rates of mortality in patients receiving high- and low-dose anticoagulation COVID-19: coronavirus disease 2019; ICU: intensive care unit; SDU: step down unit; 95% CI: 95% confidence interval; p: p-value; VTE: venous thromboembolism; RCTs: randomized controlled trials; RR: relative risk, PaO_2_: partial pressure of oxygen; FiO_2_: fraction of inspired oxygen; LMWH: low-molecular-weight heparin; IU: international unit

Study	Author	Year	Type of study	Patients	Purpose of the study	Results	Conclusion
1	Al-Banaa et al. [5]	2022	Multicenter retrospective cohort	578	To identify the relationship between doses of anticoagulation and inpatient survival among COVID-19 patients in ICU/SDU	The initiation of high-dose anticoagulant (1 mg/kg twice daily or 1.5 mg/kg daily or a continuous intravenous infusion of unfractionated heparin {UFH}) at the time of ICU/SDU admission was associated with lower in-hospital mortality compared to prophylactic dose (subcutaneous LMWH 40 mg once daily or unfractionated heparin 5000 IU twice or three times daily) (OR: 0.564, 95% CI: 0.333-0.953, p=0.032)	Timely administration of high-dose therapeutic anticoagulation was associated with lower adjusted inpatient mortality and VTE rates
2	Hoogenboom et al. [16]	2022	Retrospective observational	311	To research on the survival of critically ill COVID-19 patients who received prophylactic- or therapeutic-dose anticoagulation and assess the mortality rate concerning demographic and clinical parameters	Risk of mortality in therapeutic dose (any heparin drip or apixaban, rivaroxaban, dabigatran, or warfarin at a typical therapeutic-dose strength or enoxaparin 1 mg/kg twice daily or 1.5 mg/kg daily) versus prophylactic dose (subcutaneous heparin or enoxaparin 40 mg twice daily) (adjusted HR: 4.89, 95% CI: 1.71-14.0, p=0.003)	Therapeutically anticoagulated patients had a higher risk of death than prophylactic users
3	Farkouh et al. [22]	2022	Review: five observational studies and eight RCTs	-	The synopsis of pathology, rationale, and current evidence for the use of anticoagulation in patients with COVID-19	Three observational studies published heparin-improved survival compared to no anticoagulation. Two observational studies and five RCTs revealed pronounced benefits with a high dose	Prophylactic thromboprophylaxis significantly reduced mortality; the benefits were more distinct with high dose administration
4	Reis et al. [7]	2021	Meta-analysis: eight RCTs	5,580	To assess the efficacy and safety of different doses of anticoagulation in hospitalized patients	Intermediate-dose anticoagulation may provide little or no impact on any thrombotic event or death in moderate or severe COVID-19 (RR: 1.03; 95% CI: 0.86-1.24). Therapeutic anticoagulation may lower any thrombotic event or death in patients with moderate COVID-19 (RR: 0.64; 95% CI: 0.38-1.07) but may provide little or no impact in patients with severe disease (RR: 0.98; 95% CI: 0.86-1.12)	Moderately affected COVID-19 patients may benefit from therapeutic-dose anticoagulation but not patients with severe COVID-19
5	Kollias et al. [8]	2021	Meta-analysis: four RCTs and 20 observational studies	7,776	To estimate the risk of inpatient mortality in COVID-19 patients receiving high (intermediate or therapeutic) versus prophylactic doses of thromboprophylaxis	Pooled adjusted RR for death: intermediate versus prophylactic doses 0.56 (95% CI: 0.34-0.92). Pooled adjusted RR for death: therapeutic versus prophylactic doses 0.73 (95% CI: 0.47-1.14)	The administration of therapeutic dose did not significantly reduce mortality compared to prophylactic dose
6	Kow et al. [24]	2021	Meta-analysis: eight RCTs	5,405	To synthesize a summarized article that emphasized on RCTs for higher-intensity anticoagulation in hospitalized COVID-19 patients	Statistically not significant in the odds of mortality (pooled OR: 0.92; 95% CI: 0.71-1.19)	No statistically significant reduction in the odds of mortality in the therapeutically anticoagulated patients
7	Sholzberg et al. [17]	2021	Randomized controlled, adaptive, open-label clinical trial (28 hospitals) (RAPID RCT)	465	To examine the effects of therapeutic heparin against prophylactic heparin in moderately ill patients hospitalized with COVID-19	The occurrence of primary composite outcome in therapeutic versus prophylactic heparin (OR: 0.69, 95% CI: 0.43-1.10, p=0.12). The occurrence of the composite of all-cause mortality or any mechanical ventilation in therapeutic heparin versus prophylactic heparin (OR: 0.59, 95% CI: 0.34-1.02, p=0.06). Mortality from any cause in the therapeutic heparin and in the prophylactic heparin (OR: 0.22, 95% CI: 0.07-0.65, p=0.006)	No significant reduction in the incidence of primary outcome with therapeutic heparin; however, odds of death at 28 days were lessened
8	Giossi et al. [23]	2021	Meta-analysis: 31 observational studies and two RCTs	32,688	To determine if heparin is more effective than no anticoagulation in reducing overall mortality	The reduction of mortality with prophylactic dose (pooled HR: 0.63; 95% CI: 0.57-0.69) and with full dose (HR: 0.56; 95% CI: 0.47-0.66)	Heparin is effective in reducing mortality with both prophylactic and therapeutic doses
9	REMAP-CAP, ACTIV-4a, and ATTACC Investigators et al. [18]	2021	Open-label, adaptive, multiplatform RCT	1,098	To assess if therapeutic anticoagulation improves outcome in critically ill COVID-19 patients	Organ support-free days in patients with therapeutic dose versus prophylactic dose (adjusted proportional OR: 0.83, 95% CI: 0.67-1.03, posterior probability of futility {defined as an OR of <1.2}: 99.9%). The percentage of survivors to discharge in therapeutic versus prophylactic arms (62.7% versus 64.5%)	An initial approach with therapeutic-dose heparin did not result in a higher likelihood of survival until hospital discharge or longer free-of- organ-support days in critically ill patients with COVID-19 than routine thromboprophylaxis
10	ATTACC, ACTIV-4a, and REMAP-CAP Investigators et al. [19]	2021	Open-label, adaptive, multiplatform RCT	2,219	To assess if therapeutic anticoagulation improves outcome in noncritically ill COVID-19 patients	The probability of therapeutic dose increasing organ support-free days: 98.6% (adjusted OR: 1.27; 95% CI: 1.03-1.58, therapeutic versus prophylactic). The absolute between-group difference in survival until hospital discharge without organ support was 4% points in favor of therapeutic-dose anticoagulation (95% CI: 0.5-7.2)	Compared to routine thromboprophylaxis, an early initiation of therapeutic-dose anticoagulation with heparin enhanced the chance of survival until hospital discharge while reducing the requirement of ICU-level organ support in noncritically ill patients hospitalized with COVID-19
11	Lopes et al. [20]	2021	Pragmatic open-label, multicenter RCT: anticoagulation coronavirus (ACTION) trial	615	To compare the efficacy and safety of therapeutic with prophylactic anticoagulation in patients hospitalized with COVID-19	The number of wins was 28,899 (34.8%) in the therapeutic group and 34,288 (41.3%) in prophylactic group (win ratio: 0.86 {95% CI: 0.59-1.22}, p=0.40). Therapeutic dose: oral rivaroxaban (20 mg or 15 mg daily) for stable patients or initial subcutaneous enoxaparin (1 mg/kg twice per day) or intravenous unfractionated heparin (to achieve a 0.3-0.7 IU/mL anti-Xa concentration) for clinically unstable patients, followed by rivaroxaban to day 30. Prophylactic dose: standard in-hospital enoxaparin or unfractionated heparin	The time to death, the length of hospitalization, and the length of supplementary oxygen were not significantly different among both groups at 30 days
12	INSPIRATION Investigators et al. [3]	2021	RCT	562	The comparison of the effects of intermediate-dose with standard-dose prophylactic anticoagulation in patients with COVID-19 in the ICU	Absolute difference in the composite of adjudicated acute VTE, arterial thrombosis, treatment with extracorporeal membrane oxygenation (ECMO), or all-cause mortality within 30 days of enrollment: 1.5 (95% CI: 6.6-9.8); OR: 1.06 (95% CI: 0.76-1.48); p=0.7 (primary outcome). All-cause mortality between groups: risk difference 2.2% (95% CI: 5.9-10.3); OR 1.09 (95% CI: 0.78-1.53); p=0.5. Prophylactic dose: enoxaparin 40 mg daily. Intermediate dose: enoxaparin 1 mg/kg daily	No significant difference in the primary outcome. The regular use of intermediate-dose prophylactic anticoagulation should not be recommended in patients with COVID-19 admitted to the ICU
13	Patell et al. [12]	2021	Pooled analysis: 35 observational studies	10,857	To analyze the pooled incidence of thrombosis/bleeding in hospitalized COVID-19 patients for standard-dose, intermediate-dose, therapeutic-dose, and no pharmacologic thromboprophylaxis	The pooled incidence of overall mortality: no prophylaxis 23.1% (95% CI: 4.3-67.1); standard-dose prophylaxis 21.2% (95% CI: 17.3-25.7). The rate of all-cause mortality: intermediate-dose prophylaxis 21.0% (95% CI: 14.2-29.8) and therapeutic-dose anticoagulation 16.8% (95% CI: 15.0-18.8). Standard-dose prophylaxis: (enoxaparin 40 mg per day or equivalent dosing of other anticoagulant including other low-molecular-weight heparin or LMWH, unfractionated heparin, or direct oral anticoagulant {DOAC}). Intermediate-dose prophylaxis (weight-adjusted, double-dose prophylaxis or any dosage that is greater than the standard dose and lower than the therapeutic-dose anticoagulants). Therapeutic-dose anticoagulants: enoxaparin 1 mg/kg twice daily or 1.5 mg/kg once daily or equivalent doses of other anticoagulants including other LMWH, unfractionated heparin, or DOAC	The rate of reduction in mortality was observed by increasing the doses of anticoagulation
14	Ionescu et al. [6]	2020	Retrospective multicenter cohort study	3,480	To assess the impact of different anticoagulant doses on survival in COVID-19 patients	The probability of survival at 25 days: therapeutic (57.5%) versus prophylactic dose (50.7%). Inpatient mortality: no prophylaxis (11.4%), standard-dose prophylaxis (10.8%), and therapeutic anticoagulation (23.6%) (p<0.001). Compared to no prophylaxis, standard prophylaxis was associated with a 65% reduction in mortality risk (HR: 0.35; 95% CI: 0.22-0.54) and therapeutic anticoagulation with 86% reduction (HR: 0.14; 95% CI: 0.05-0.23). Therapeutic dose: (a) intravenous unfractionated heparin (UFH) with at least one documented activated partial thromboplastin time in the anticoagulation range (≥45 seconds), (b) subcutaneous enoxaparin at doses of 1 mg/kg twice daily or 1.5 mg/kg once daily (while allowing for dose adjustment based on creatinine clearance), (c) intravenous argatroban infusion, (d) subcutaneous fondaparinux at doses of 5-10 mg once daily (weight-based dosing), or (e) oral anticoagulants (warfarin, apixaban, rivaroxaban, and dabigatran) prescribed prior to and continued throughout hospitalization. Prophylactic dose: (1) subcutaneous injection of UFH at doses of 5,000 units twice or three times daily; (2) subcutaneous enoxaparin injection at doses of 30-40 mg once daily; or (3) subcutaneous fondaparinux at a dose of 2.5 mg once daily	A dose-dependent effect was observed. Higher doses of anticoagulation were associated with a lower risk of death in patients hospitalized due to COVID-19
15	Kamel et al. [2]	2020	Meta-analysis: 16 retrospective cohorts, three prospective cohorts, and one case‐control study	-	To identify the association between different doses of anticoagulation and mortality in patients with COVID-19	The association between antithrombotic therapy and mortality was statistically significant (RR: 0.56, 95% CI: 0.36-0.92, p=0.02). Greater risk of mortality in prophylactic-dose participants compared to therapeutic-dose counterparts (RR: 1.58, 95% CI: 1.34-1.87, p<0.001)	Both prophylactic-dose and therapeutic-dose antithrombotic therapies might decrease the risk of mortality in COVID-19 patients
16	Lemos et al. [21]	2020	Randomized, open-label, phase II study (HESACOVID)	20	To evaluate whether therapeutic anticoagulation enhances gas exchange and reduces the demand for mechanical ventilation in severe COVID-19 patients compared to conventional dosage	The therapeutic dosing arm significantly increased the PaO_2_/FiO_2_ overtime: 163 (95% CI: 133-193) at baseline, 209 (95% CI: 171-247) after seven days, and 261 (95% CI: 230-293) after 14 days (p=0.0004)	Therapeutic anticoagulation improved gas exchange and decreases the dependence on mechanical ventilation in severe COVID-19. The study was not powerful enough to compare mortality

Hiking Up of Bleeding Events in Full-Dose Anticoagulation

Although the general bleeding rate was more notable in patients receiving higher anticoagulation doses, the risk of severe bleeding events was similar in both groups [5]. The meta-analysis of Reis et al. showed that therapeutic-dose anticoagulation might promote serious hemorrhage within 30 days relative to routine thromboprophylaxis, regardless of disease severity [7]. These findings coincided with analyses of several other studies that highlighted that higher-dose thromboprophylaxis was associated with a higher occurrence of major bleeding complications than routine thromboprophylaxis [3,6,17-25]. Kollias et al. analyzed that the incidence of major bleeding events was significantly associated with therapeutic-dose thromboprophylaxis. However, the result was invalid for the intermediate-dose prophylaxis group [8]. Moreover, different trends were observed in the INSPIRATION trial that did not detect a significant difference in both major and clinically relevant nonmajor bleeding events between intermediate and standard prophylactic-dose regimens. Similarly, a pooled analysis led by Patell et al. disclosed that hemorrhagic rates for participants receiving intermediate- or therapeutic-dose anticoagulation were comparable to those receiving standard-dose anticoagulation [12]. Sholzberg et al. also reported a low risk of major bleeding in patients allocated to therapeutic-dose anticoagulation [17]. With an assumed control risk of 0.014 (the frequency of significant bleeding in the control group was 14 per 1,000), approximately 90 patients would require higher-intensity anticoagulation dosing to experience one major bleeding event [20]. Table 4 recapitulates the studies discussing the bleeding events associated with prophylactic- and therapeutic-dose anticoagulation.

**Table 4:** Effects of high- and low-dose antithrombosis on hemorrhagic events COVID-19: coronavirus disease 2019; ICU: intensive care unit; SDU: step down unit; p: p-value; RCTs: randomized controlled trials; RR: relative risk, 95% CI: 95% confidence interval; ∞: infinity; IU: international unit

Study	Author	Year	Type of study	Patients	Purpose of the study	Results	Conclusion
1	Al-Banaa et al. [5]	2022	Multicenter retrospective cohort	578	To identify the relationship between doses of anticoagulation and inpatient survival among COVID-19 patients in ICU/SDU	The rate of major hemorrhage was 1.6% (n=7) in high-dose versus 0.8% (n=1) in low-dose anticoagulation (p=0.45). No significant difference in the unadjusted rate of any bleeding event (5.1%, n=23) with high dose and (3.1%, n=4) with low dose (p=0.32). Therapeutic dose: 1 mg/kg twice daily or 1.5 mg/kg daily or a continuous intravenous infusion of unfractionated heparin. Prophylactic dose: subcutaneous low-molecular-weight heparin (LMWH) 40 mg once daily or unfractionated heparin (UFH) 5000 IU twice or three times daily	Despite the greater incidence of overall bleeding rate with a higher dose, the rate of major hemorrhagic complications is considerably low in both groups
2	Farkouh et al. [22]	2022	Review: five observational studies and eight RCTs	-	The synopsis of pathology, rationale, and current evidence for the use of anticoagulation in patients with COVID-19	One RCT reported the incidence of major bleeding: 1.9% (therapeutic dosage) versus 0.9% (prophylactic dosage). One RCT stated a rising trend of major bleeding in full-dose receivers	Serious hemorrhage was associated with high-dose administration
3	Reis et al. [7]	2021	Meta-analysis: eight RCTs	5,580	To assess the efficacy and safety of different doses of anticoagulation in hospitalized patients	The risk of major bleeding was increased in both intermediate dosing (RR: 1.48; 95% CI: 0.53-4.15) and therapeutic dosing (RR: 1.78; 95% CI: 1.15-2.74)	Major bleeding events were greater with intermediate and therapeutic anticoagulation irrespective of disease severity
4	Kollias et al. [8]	2021	Meta-analysis: four RCTs and 20 observational studies	7,776	To estimate the risk of inpatient mortality in COVID-19 patients receiving high (intermediate or therapeutic) versus prophylactic doses of thromboprophylaxis	Pooled adjusted RR for major bleeding events: intermediate versus prophylactic doses 1.63 (95% CI: 0.79-3.37). Pooled adjusted RR for major bleeding events: therapeutic versus prophylactic doses 3.32 (95% CI: 2.51-4.40)	The incidence of major bleeding events was significantly associated with therapeutic-dose thromboprophylaxis. However, the result was not significant for the intermediate-dose prophylaxis group
5	Kow et al. [24]	2021	Meta-analysis: eight RCTs	5,405	To synthesize a summarized article that emphasized RCTs for higher-intensity anticoagulation in hospitalized COVID-19 patients	Statistically significant increase in odds of the development of serious hemorrhage (pooled OR: 1.81; 95% CI: 1.20-2.72) with intermediate/therapeutic anticoagulation	Higher doses were associated with an elevated risk of major bleeding
6	Sholzberg et al. [17]	2021	Randomized controlled, adaptive, open-label clinical trial (28 hospitals) (RAPID RCT)	465	To examine the effects of therapeutic heparin against prophylactic heparin in moderately ill patients hospitalized with COVID-19	Odds of major hemorrhage with therapeutic heparin and prophylactic heparin (OR: 0.52, 95% CI: 0.09-2.85, p=0.69)	Increased risk of bleeding was associated with high dose of heparin
7	Giossi et al. [23]	2021	Meta-analysis: 31 observational studies and two RCTs	32,688	To determine if heparin is more effective than no anticoagulation in reducing overall mortality	No significant increase in bleeding risk with full-dose heparin versus no treatment (OR: 0.81, 95% CI: 0.66-1.0; OR: 1.55, 95% CI: 0.982.44, respectively). Greater risk of major bleeding with full dose (OR: 2.01; 95% CI: 1.14-3.53) versus prophylactic dose	Therapeutic dose was associated with a higher risk of bleeding
8	REMAP-CAP, ACTIV-4a, and ATTACC Investigators et al. [18]	2021	Open-label, adaptive, multiplatform RCT	1,098	To assess if therapeutic anticoagulation improves outcome in critically ill COVID-19 patients	Major bleeding in therapeutic versus prophylactic dose (3.8% versus 2.3%)	The occurrence of major bleeding was higher in therapeutic anticoagulation
9	ATTACC, ACTIV-4a, and REMAP-CAP Investigators et al. [19]	2021	Open-label, adaptive, multiplatform RCT	2,219	To assess if therapeutic anticoagulation improves outcome in noncritically ill COVID-19 patients	Major bleeding in therapeutic versus prophylactic dose (1.9% versus 0.9%)	The occurrence of major bleeding was higher in patients receiving therapeutic anticoagulation
10	Lopes et al. [20]	2021	Pragmatic open-label, multicenter RCT: anticoagulation coronavirus (ACTION) trial	615	To compare the efficacy and safety of therapeutic with prophylactic anticoagulation in patients hospitalized with COVID-19	The occurrence of major or clinically significant nonmajor bleeding in therapeutic versus prophylactic dose (RR: 3.64; 95% CI: 1.61-8.27). Therapeutic dose: oral rivaroxaban (20 mg or 15 mg daily) for stable patients or initial subcutaneous enoxaparin (1 mg/kg twice per day) or intravenous unfractionated heparin (to achieve a 0.3-0.7 IU/mL anti-Xa concentration) for clinically unstable patients, followed by rivaroxaban to day 30. Prophylactic dose: standard in-hospital enoxaparin or unfractionated heparin	Therapeutic anticoagulation increased the risk of bleeding compared to prophylactic arm
11	INSPIRATION Investigators [3]	2021	RCT	562	The comparison of the effects of intermediate-dose with standard-dose prophylactic anticoagulation in patients with COVID-19 in the ICU	Major bleeding in intermediate versus prophylactic dose: risk difference 1.1% (one-sided 97.5% CI: ∞-3.4); OR 1.83 (one-sided 97.5% CI: 0.00-5.93) (p for noninferiority of >0.99). Prophylactic dose: enoxaparin 40 mg daily. Intermediate dose: enoxaparin 1 mg/kg daily	No statistical differences in the occurrence of major bleeding between different doses of antithrombosis
12	Tacquard et al. [25]	2021	Retrospective observational study: eight French ICUs	-	To investigate the incidence of thrombotic events and bleeding in severely ill COVID-19 patients and their association with prophylactic anticoagulation dosages	Exposure to higher prophylactic dosing was not associated with increased bleeding risk compared to standard dosing within 24 hours before the event (HR: 0.63; 95% CI: 0.28-1.44) or with cumulative exposure (HR: 1.11; 95% CI: 0.70-1.75)	High-dose anticoagulation was not associated with increased risk of bleeding
13	Patell et al. [12]	2021	Pooled analysis: 35 observational studies	10,857	To analyze the pooled incidence of thrombosis/bleeding in hospitalized COVID-19 patients for standard-dose, intermediate-dose, therapeutic-dose, and no pharmacologic thromboprophylaxis	No significant difference in pooled bleeding event rates (n=393) between therapeutic-dose and standard-dose prophylaxis (6.3 versus 1.7%; p=0.083). Standard-dose prophylaxis: enoxaparin 40 mg per day or equivalent dosing of other anticoagulant including other low-molecular-weight heparin or LMWH, unfractionated heparin, or direct oral anticoagulant (DOAC). Intermediate-dose prophylaxis (weight-adjusted, double-dose prophylaxis or any dosage that is greater than the standard dose and lower than the therapeutic-dose anticoagulants). Therapeutic-dose anticoagulants: enoxaparin 1 mg/kg twice daily or 1.5 mg/kg once daily or equivalent doses of other anticoagulants including other LMWH, unfractionated heparin, or DOAC	The pooled bleeding event rates were not significantly higher in full-dose anticoagulation
14	Ionescu et al. [6]	2020	Retrospective multicenter cohort study	3,480	To assess the impact of different anticoagulant doses on survival in COVID-19 patients	The occurrence of major bleeding: no anticoagulation (5.5%), prophylactic anticoagulation (2.2%), and therapeutic anticoagulation (8.1%). Therapeutic dose: (a) intravenous unfractionated heparin (UFH) with at least one documented activated partial thromboplastin time in the anticoagulation range (≥45 seconds), (b) subcutaneous enoxaparin at doses of 1 mg/kg twice daily or 1.5 mg/kg once daily (while allowing for dose adjustment based on creatinine clearance), (c) intravenous argatroban infusion, (d) subcutaneous fondaparinux at doses of 5-10 mg once daily (weight-based dosing), or (e) oral anticoagulants (warfarin, apixaban, rivaroxaban, and dabigatran) prescribed prior to and continued throughout hospitalization. Prophylactic dose: (1) subcutaneous injection of UFH at doses of 5,000 units twice or three times daily; (2) subcutaneous enoxaparin injection at doses of 30-40 mg once daily; or (3) subcutaneous fondaparinux at a dose of 2.5 mg once daily	Major bleeding events occurred more frequently in therapeutic dosing strategy

Slimming of Thromboembolic Events With Therapeutic-Dose Anticoagulation

A systematic review and meta-analysis highlighted that the total incidence of venous thromboembolism (VTE) among COVID-19 inpatients was reported to be 17% (95% CI: 13.4-20.9), with variations depending on research design and method ascertainment; ICU patients had a fourfold greater incidence rate than those in non-ICU settings (28% versus 7%) [11]. Patell et al. supplemented these findings by stating that severely ill patients have an elevated risk of thrombosis, partly due to a combination of prothrombotic risk factors such as chronic immobility and hyperinflammatory conditions [12]. Hasan et al. stated that prophylactic LMWH was associated with subtherapeutic anti-factor Xa levels in critically ill COVID-19 patients [26]. The author also mentioned that monitoring anti-factor Xa levels in patients on UFH was associated with a better achievement of therapeutic anticoagulation than monitoring activated partial thromboplastin time (APTT). In patients receiving high doses of UFH to reach the goal APTT, the risks of life-threatening hemorrhagic events might be higher without monitoring antithrombotic activity via an anti-factor Xa assay. His meta-analysis reported a lower prevalence of VTE in patients allocated to the mixed anticoagulation approach (prophylactic and therapeutic) compared to patients allocated to prophylactic anticoagulation only. Hasan et al. hypothesized that a lower occurrence of VTE in the former arm might be due to a higher rate of the achievement of desired anti-factor Xa level from the administration of therapeutic-dose anticoagulation [26]. Similar to Farkouh et al. [22], Al-Banaa et al. [5] agreed that full-dose anticoagulation was correlated with a decrease in thromboembolism. Reis et al. analyzed that the risk of any thrombotic events may be lower in therapeutically anticoagulated participants in contrast to prophylactically anticoagulated counterparts independent of disease severity [7]. In addition, the meta-analysis of Kow et al. reported that the reduction in the risk of thrombotic complications with intermediate or therapeutic anticoagulation was statistically significant compared to standard thromboprophylaxis [24]. Tacquard et al. disclosed that cumulative exposure to higher-dose antithrombotic therapy was significantly associated with a decreased risk of thrombotic events, highlighting the potential benefits of a higher-dose anticoagulant regimen in severe COVID-19 patients [25].

Nevertheless, the ACTION and INSPIRATION trials did not detect a significant difference in the risk of VTE between intermediate-dose and standard-dose strategies [3,20]. Similarly, the REMAP-CAP, ACTIV-4a, and ATTACC Investigators and the RAPID trial reported that the difference in the incidence of thromboembolic events in therapeutic and prophylactic groups was not statistically significant [16-18]. Patell et al. found similar results in the total VTE rate among prophylactic-dose, intermediate-dose, and therapeutic-dose thromboprophylaxis strategies [12]. Although conventional pharmacologic thromboprophylaxis is indicated in hospitalized patients, a number of expert organizations have supported raising anticoagulant doses in patients with severe symptoms of COVID-19. The French Working Group on Perioperative Hemostasis and the French Study Group on Thrombosis and Hemostasis have postulated gradually increasing the dose of anticoagulants based on thrombotic risk factors such as obesity, high oxygen demand, the need for mechanical ventilation, and biomarkers of systemic inflammation or hypercoagulability albeit no supporting evidence [25]. Furthermore, Kow et al. demonstrated that the estimated number needed for higher dosing of thromboprophylaxis to prevent one VTE event would be 37 assuming a control risk of 0.062 [24]. Table 5 includes the studies comparing the occurrence of VTE with various doses of anticoagulation.

**Table 5:** Comparison of thromboembolic events with different doses of thromboprophylactic therapy COVID-19: coronavirus disease 2019; ICU: intensive care unit; SDU: step down unit; RCTs: randomized controlled trials; RR: relative risk; 95% CI: 95% confidence interval; p: p-value; IU: international unit

Study	Author	Year	Type of study	Patients	Purpose of the study	Results	Conclusion
1	Al-Banaa et al. [5]	2022	Multicenter retrospective cohort	578	To identify the relationship between doses of anticoagulation and inpatient survival among COVID-19 patients in ICU/SDU	Fewer thrombotic events were inspected in therapeutic anticoagulation (6.4% versus 10.4%). Therapeutic dose: 1 mg/kg twice daily or 1.5 mg/kg daily or a continuous intravenous infusion of unfractionated heparin. Prophylactic dose: subcutaneous low-molecular-weight heparin (LMWH) 40 mg once daily or unfractionated heparin 5000 IU twice or three times daily	Prophylactic dosing was associated with more venous thromboembolisms (VTEs)
2	Farkouh et al. [22]	2022	Review: five observational studies and eight RCTs	-	The synopsis of pathology, rationale, and current evidence for the use of anticoagulation in patients with COVID-19	One RCT found a reduction in thromboembolism with full dose versus low or intermediate doses (RR: 0.37, 95% CI: 0.21-0.66, p<0.001). One RCT concluded no significant difference between intermediate- and prophylactic-dose arms (OR: 1.06, 95% CI: 0.76-1.48, p=0.70). One RCT detected only subtle differences between groups with in-hospital major thrombotic events (1.1% in therapeutic dose versus 2.1% in prophylactic dose)	Full-dose anticoagulation was associated with a decrease in thromboembolic events compared to prophylactic or intermediate dose
3	Reis et al. [7]	2021	Meta-analysis: eight RCTs	5,580	To assess the efficacy and safety of different doses of anticoagulation in hospitalized patients	Compared to conventional thromboprophylaxis, therapeutic anticoagulation may reduce the risk of any thrombotic event in 28 days (RR: 0.58; 95% CI: 0.45-0.74; 4669 participants, six studies, moderate-certainty evidence)	COVID-19 patients may benefit from therapeutic-dose anticoagulation
4	Kollias et al. [8]	2021	Meta-analysis: four RCTs and 20 observational studies	7,776	To estimate the risk of inpatient mortality in COVID-19 patients receiving high (intermediate or therapeutic) versus prophylactic doses of thromboprophylaxis	Pooled adjusted RR for VTE: intermediate versus prophylactic doses 0.84 (95% CI: 0.54-1.31). Pooled adjusted RR for VTE: therapeutic versus prophylactic doses 1.13 (95% CI: 0.52-2.48)	No significant difference in the occurrence of VTE with high versus low doses
5	Kow et al. [24]	2021	Meta-analysis: eight RCTs	5,405	To synthesize a summarized article that emphasized on RCTs for higher-intensity anticoagulation in hospitalized COVID-19 patients	Significantly decreased the odds of the development of thrombotic events (pooled OR: 0.66; 95% CI: 0.45-0.98)	The administration of intermediate/therapeutic anticoagulation reduced the risk of VTEs
6	Sholzberg et al. [17]	2021	Randomized controlled, adaptive, open-label clinical trial (28 hospitals) (RAPID RCT)	465	To examine the effects of therapeutic heparin against prophylactic heparin in moderately ill patients hospitalized with COVID-19	Venous thromboembolism in therapeutic heparin versus prophylactic heparin (OR: 0.34, 95% CI: 0.07-1.71, p=0.19)	The occurrence of VTE was low in both groups
7	REMAP-CAP, ACTIV-4a, and ATTACC Investigators et al. [18]	2021	Open-label, adaptive, multiplatform RCT	1,098	To assess if therapeutic anticoagulation improves outcome in critically ill COVID-19 patients	Major thrombotic events in therapeutic versus prophylactic dose (6.4% versus 10.4%)	No significant difference was observed in both strategies
8	ATTACC, ACTIV-4a, and REMAP-CAP Investigators et al. [19]	2021	Open-label, adaptive, multiplatform RCT	2,219	To assess if therapeutic anticoagulation improves outcome in noncritically ill COVID-19 patients	Major thrombotic events in therapeutic versus prophylactic dose (8% versus 9.9%)	The incidence of thromboembolism was similar in both groups
9	Lopes et al. [20]	2021	Pragmatic open-label, multicenter RCT: anticoagulation coronavirus (ACTION) trial	615	To compare the efficacy and safety of therapeutic with prophylactic anticoagulation in patients hospitalized with COVID-19	The incidence of the composite of venous thromboembolism, myocardial infarction, stroke, systemic embolism, or major adverse limb events in therapeutic versus prophylactic (RR: 0.75, 95% CI: 0.45-1.26, p=0.32). Therapeutic dose: oral rivaroxaban (20 mg or 15 mg daily) for stable patients or initial subcutaneous enoxaparin (1 mg/kg twice per day) or intravenous unfractionated heparin (to achieve a 0.3-0.7 IU/mL anti-Xa concentration) for clinically unstable patients, followed by rivaroxaban to day 30. Prophylactic dose: standard in-hospital enoxaparin or unfractionated heparin	No statistical difference in the incidence of thrombotic events between groups at 30 days
10	INSPIRATION Investigators [3]	2021	RCT	562	The comparison of the effects of intermediate-dose and standard-dose prophylactic anticoagulation in patients with COVID-19 in the ICU	The risk of VTE difference: 0.2% (95% CI: 3.2-2.7); OR: 0.93 (95% CI: 0.37-2.32) (p=0.94). Prophylactic dose: enoxaparin 40 mg daily. Intermediate dose: enoxaparin 1 mg/kg daily	Differences in risk of VTEs were not significant
11	Tacquard et al. [25]	2021	Retrospective observational study	Eight French ICUs	To investigate the incidence of thrombotic events and bleeding in severely ill COVID-19 patients and their association with prophylactic anticoagulation dosages	Reduced risk of thrombotic complications in high dosing group (HR: 0.81; 95% CI: 0.66-0.99) without increasing the risk of bleeding. Prolonged exposure to high-dose prophylactic anticoagulation was also significantly associated with a reduction in the risk of thromboembolism (HR: 0.79, 95% CI: 0.65-0.95, p=0.014)	Cumulative exposure to high-dose antithrombosis was more likely to diminish the risk of thrombosis
12	Patell et al. [12]	2021	Pooled analysis: 35 observational studies	10,857	To analyze the pooled incidence of thrombosis/bleeding in hospitalized COVID-19 patients for standard-dose, intermediate-dose, therapeutic-dose, and no pharmacologic thromboprophylaxis	The pooled incidence rates of total VTE (n=4,685): no prophylaxis 41.9% (95% CI: 28.1-57.2), standard-dose prophylaxis 19.8% (95% CI: 13.2-28.6), intermediate-dose prophylaxis 11.9% (95% CI: 4.3-28.6), and therapeutic-dose anticoagulation 10.5% (95% CI: 4.2-23.8). The pooled incidence rates of arterial thrombosis (n=1,464): no prophylaxis 11.3% (95% CI: 5.2-23.0), standard-dose prophylaxis 2.5% (95% CI: 1.4-4.3), intermediate-dose prophylaxis 2.1% (95% CI: 0.5-7.7), and therapeutic-dose anticoagulation 1.3% (95% CI: 0.2-8.8) (p=0.009). Standard-dose prophylaxis: enoxaparin 40 mg per day or equivalent dosing of other anticoagulants including other low-molecular-weight heparin or LMWH, unfractionated heparin, or direct oral anticoagulant (DOAC). Intermediate-dose prophylaxis (weight-adjusted, double-dose prophylaxis or any dosage that is greater than the standard dose and lower than the therapeutic-dose anticoagulants). Therapeutic-dose anticoagulants: enoxaparin 1 mg/kg twice daily or 1.5 mg/kg once daily or equivalent doses of other anticoagulants including other LMWH, unfractionated heparin, or DOAC	Doses of anticoagulants were inversely associated with incidence rates of both venous and arterial thromboembolisms
13	Hasan et al. [26]	2020	Meta-analysis: 12 studies	-	To systematically review the available evidence regarding the anticoagulation approach to prevent venous thromboembolism	The pooled prevalence of VTE among ICU patients in all studies was 31% (95% CI: 20-43; I2: 92%). Subgroup pooled analysis: studies of patients on prophylactic anticoagulation alone showed a pooled prevalence of VTE of 38% (95% CI: 10-70; I2: 96%). Studies of patients on mixed therapeutic and prophylactic anticoagulation reported a pooled prevalence of VTE of 27% (95% CI: 17-40; I2: 89%)	The lower prevalence of VTE was seen in studies that reported mixed therapeutic and prophylactic anticoagulation. Prophylactic LMWH dosing was associated with subtherapeutic anti-factor Xa levels in critically ill patients. Anti-factor Xa assay was encouraged

Limitations

Our systematic review has a number of limitations. We obtained our data from only three databases (PubMed, PMC, and MEDLINE) and retrieved the records providing free access to full texts published in English. There is a chance that unincluded research may provide different results. We did not have a statistician to analyze the included data; therefore, we had to rely on the presented results.

## Conclusions

The majority of studies included in our systematic review indicate that therapeutic-dose anticoagulation was associated with a lower rate of thromboembolism and an increased risk of bleeding. However, whether a high-dose strategy can reduce mortality remains inconclusive. Many publications have different interpretations of the superiority of therapeutic anticoagulation, while the certainty of evidence from available data is still low. Globalized, large-scale randomized controlled trials powerful enough to detect statistically and clinically significant differences between high and low doses are required to clarify the ideal dosage and timing of anticoagulation.
